# The Role of Parental Concerns in the Recognition of Sepsis in Children: A Literature Review

**DOI:** 10.3389/fped.2019.00161

**Published:** 2019-05-03

**Authors:** Amanda Harley, Jos M. Latour, Luregn J. Schlapbach

**Affiliations:** ^1^Paediatric Critical Care Research Group, Child Health Research Centre and Faculty of Medicine, The University of Queensland, Brisbane, QLD, Australia; ^2^Paediatric Intensive Care Unit, Queensland Children's Hospital, Children's Health Queensland, Brisbane, QLD, Australia; ^3^School of Nursing, The University of Queensland, Brisbane, QLD, Australia; ^4^Faculty of Health and Human Sciences, School of Nursing and Midwifery, University of Plymouth, Plymouth, United Kingdom; ^5^Department of Paediatrics, Bern University Hospital, Inselspital, University of Bern, Bern, Switzerland

**Keywords:** child, concern, diagnosis, infection, parent, recognition, sepsis, septic shock

## Abstract

**Background:** Sepsis is a time critical disease and outcomes strongly depend on time to initiation of appropriate treatment in hospital. A range of studies have assessed sepsis recognition in hospital settings, whereas little is known about sepsis recognition in the community. The decision-making of parents in seeking medical care may substantially impact survival of children with sepsis. An improved understanding of the parental perspective in recognizing sepsis is urgently needed to inform the design of education campaigns and consideration of using parental concerns as a trigger in sepsis screening tools.

**Aim:** To review the literature on parental concerns in the diagnosis of sepsis in children.

**Methods:** A literature review on parental concerns in pediatric sepsis was performed accessing publications in PubMed, CINAHL and Medline published between 1990 and 2018. In addition, we compared guidelines and online institutional sepsis recognition tools and assessed whether parental concerns were used for screening.

**Results:** Out of 188 articles reviewed, 11 met the criteria. One article was found prospectively assessing the diagnostic performance of parental concern in children evaluated for infection, indicating high positive (16.4) and negative likelihood ratio (0.23) for sepsis/meningitis in presence of parental concerns. The role of parental concern was listed as a sign assisting recognition of sepsis in four studies reporting original data, and six reviews commented on parental concern listed as a factor upon diagnosis of sepsis. When comparing selected examples of institutional sepsis pathways available online, parental concern was variably listed as a criterion to prompt evaluation for sepsis.

**Conclusions:** Despite some guidelines emphasizing the role of parental concern in recognizing sepsis, there is a paucity of data in the field. An improved understanding of whether parental concerns adds diagnostic value to sepsis recognition at acceptable sensitivity and specificity is urgently needed. Future prospective studies should assess whether including parental concerns in sepsis screening tools benefits the assessment resulting in early diagnosis and treatment of children with sepsis.

## Introduction

Sepsis represents a leading cause of global childhood mortality (1–3). In response to the recent resolution by the World Health Organization recognizing sepsis as a priority in healthcare(4, 5), several national and regional healthcare systems have implemented sepsis pathways to improve recognition and early treatment of sepsis in hospital settings (6). Sepsis in children remains a time critical disease and the majority of deaths and multi-organ dysfunction occur within the first 48 h of admission (7–10), highlighting the relevance of timely intervention. While interventional trials in children and adults have failed to result in reduced mortality (11–13), observational studies have consistently indicated that time to sepsis treatment strongly impacts on sepsis survival (14–18).

Physiologic criteria, early warning tools, and electronic health-record based trigger tools have been reported to improve the recognition of children with severe bacterial infections, and sepsis (19–23). Currently used sepsis recognition tools yield high sensitivity but mostly at the expense of poor specificity, given that most infectious illnesses in children manifest with fever, tachycardia, and tachypnea (24, 25). Inaccurate sepsis diagnosis may lead to unnecessary antibiotic therapy, hospitalization, and missed alternative diagnoses. The importance of balancing the need for rapid sepsis recognition vs. potential adverse effects on patients, healthcare resource use and antimicrobial resistance related to overtreatment is becoming increasingly recognized (26, 27) implicating an urgent need for rigorous studies on sepsis recognition.

Importantly, sepsis starts most commonly in the community and the decision and timing of parents in seeking medical care for children is likely to contribute to severity upon presentation and sepsis-related outcomes. Root cause analyses after fatal sepsis outcomes in children often report on recurrent presentations to hospital (16) and anecdotal data reveals parents in such cases often indicated concerns that “this disease is different” suggesting parents may have sensed the potential severity of the disease prior to the recognition of sepsis by clinicians. Increasing parental education has been demonstrated to reduce infant mortality due to infections in low income settings (28, 29). Yet the potential value of including parental assessment in discriminating children with mild infections from sepsis has received little attention. The capacity of parents to assess whether a disease presents differently to previous common febrile illnesses could potentially result in improved diagnostic accuracy of sepsis assessment.

We therefore aimed to review the literature on parental concerns in recognizing sepsis in children, with particular focus on studies reporting on diagnostic accuracy. In addition, we searched whether online available institutional sepsis screening tools include parental concern as a trigger for recognition or escalation.

## Methods

### Objectives

To review the literature on parental concern in the diagnosis of sepsis in children.

### Eligibility Criteria

The Population-Intervention-Control-Outcome-Study design (PICOS) approach was applied and guided the literature review focusing on: (P) pediatric age groups of <18 years of age; with (I) parental concern utilized as an assessment tool; (C) control consisting of standard diagnostic approach without including parental concern as a diagnostic tool; (O) diagnosis of sepsis and diagnostic accuracy of sepsis diagnosis as outcomes; and (S) both quantitative and qualitative original research, case reports, editorials/viewpoints, guidelines, and reviews included.

### Search Strategy

Three strategies for data collection were utilized: First, a comprehensive search for published literature through international databases was performed. Second, we manually searched reference lists from articles identified through the database search. Third, we considered additional articles identified as relevant by the authors. Publications were accessed in three literature databases: MEDLINE, CINAHL and PUBMED. Search terms used included: “Concern” OR “worry” OR “fear,” AND “infant” OR “pediatric” OR “pediatric” OR “child” OR “neonate” OR “childhood,” AND “sepsis” OR “septic” OR “severe sepsis” OR “septic shock” OR “bacteremia” OR ”severe infection” OR “systemic inflammatory response syndrome,” AND “parent” OR “family” OR “caregiver” OR “mother” OR “father” (Supplementary Table 1).

Studies were considered if they were published as full text in the English language between January 1st 1990 and September 1st 2018. Duplicate references were removed manually. Original research, case reports, editorials/viewpoints, guidelines, and reviews were considered. The initial title and abstract screen for further review was conducted by two authors (AH, LS). Articles for full text review were selected by applying the search terms to the titles and abstracts of articles.

We then performed a two-stage review of full text articles. In the first stage, we searched articles that provided original data on parental concerns in pediatric sepsis. We considered publications that defined sepsis, including septic shock and severe sepsis, according to the 2005 International Pediatric Sepsis Definition Conference (30), the American College of Chest Physicians (31), or adaptations from the recent Sepsis-3 criteria (24, 32). We included articles which reported on parental concern for children below 18 years as part of diagnostic assessment for patients presenting with sepsis or severe infection. Given the low yield of only one article meeting the PICOS criteria, we included studies reporting on severe infections, and bacteraemia. In the second stage, we searched full-text articles that reported on the use or importance of parental concerns in pediatric sepsis without providing original data.

Articles were excluded if full text was unavailable in English was unavailable. All full text articles identified underwent review by two independent investigators (AH, LJS). Clarification for inclusion was resolved by discussion. Due to the paucity of data reporting on diagnostic accuracy, sensitivity, specificity, and negative and positive predictive value in relation to parental concern, a meta-analysis could not be performed.

### Inclusion of Parental Concerns in Pediatric Sepsis Pathways

In order to compare examples of pediatric sepsis pathways in relation to utilization of parental concern, we selected published or online accessible pediatric sepsis pathways available in English language which were published in the past 5 years (date of updated search September 1st 2018). We limited the search to published international pediatric sepsis guidelines, and to pathways of jurisdictions which had previously reported on state- or nationwide sepsis campaigns (14, 33–35). This resulted in examples of pathways from three continents (North America, Europe and Oceania). These examples were selected to show different approaches to the recognition of sepsis in children. Pathways were then searched for the presence of a field listing parental concern as a risk factor or warning sign for sepsis. We considered terms such as “concern” (OR “worry” OR “fear”), and “parent” (OR “family” OR “caregiver” OR “mother/maternal” OR “father/paternal”).

## Results

### Study Selection

**Figure 1:**
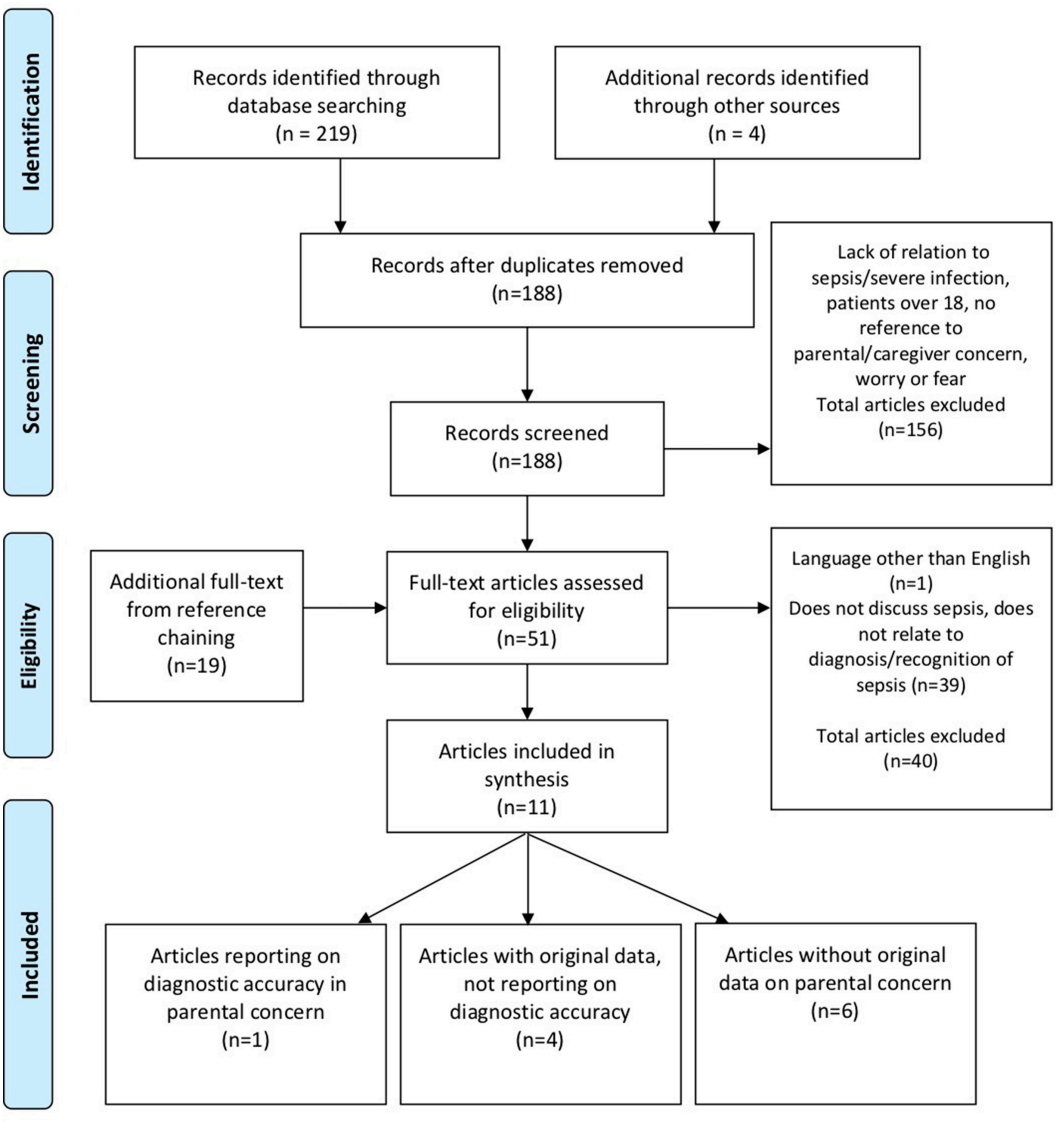
PRISMA flow diagram.

The search of the databases yielded 219 results and an additional four articles through other sources. After exclusion of duplicates and records in languages other than English, 188 remained (Figure 1). Abstract review by two assessors excluded 156 articles as the records were not reporting on sepsis diagnosis pertinent to parental concerns and children. Reference chaining identified a further 19 articles. In total, 51 Articles were selected for full-text review by two assessors. Of these, a further 40 were excluded because of one study in non-English language (36), and 39 because they did not report on parental concern in relation to recognition of sepsis. We included 11 articles in the review, of which five reported original data, out of which one only reported on diagnostic accuracy. Six articles were reviews reporting on parental concern without original data.

### Study Characteristics

Only one study reported original data on diagnostic accuracy of parental assessment in relation to sepsis (Table 1). Van den Bruel performed a prospective multicenter study in primary care settings including 3981 children which presented to 121 physicians (General practitioners, pediatricians, and emergency physicians) (37). The study was designed to assess diagnostic accuracy of several clinical features including physiological variables, clinician perception that “something is wrong,” and parental concern. Parental concern was defined as the parental perception or statement that the “disease is different.” Classification and regression tree analysis was performed to define the best performing criteria and criteria combination for clinical practice. Out of the 3981 included children (mean age 5.0 years, range = 0.02–16.9 years), 31 (0.78%) had a serious bacterial infection and 9 (0.22%) were diagnosed with sepsis and/or meningitis. Presence of parental concern that the disease is different was associated with an odds ratio (OR) of 70.5 (95%-CI 14.5 to 341.4) for sepsis/meningitis in the decision tree model, and a sensitivity of 77.8%, specificity of 95.1%, positive predictive value (PPV) of 3.6%, negative predictive value (NPV) of 100%, positive likelihood ratio (PLR) of 16.4, and negative likelihood ratio (NLR) of 0.23. In comparison, the assessment by the treating physician that “something is wrong” was associated with sepsis with an OR of 268 (33–2,163) and a sensitivity of 88.9% and specificity of 97.1%. The sensitivity of parental concern to capture any serious bacterial infection was 46.4%, and the according specificity was 96.8%, PPV 9.5%, NPV 99.6%, PLR 14.35 and NLR 0.55, respectively.

**Table 1:** Original studies reporting on diagnostic accuracy of parental concern in diagnosis of severe infection and sepsis.

**References and country of enrolment**	**Study design**	**Patients**	**Inclusion criteria**	**Outcomes in relation to parental concern**	**Comments**
Van den Bruel et al. (37), Belgium	Prospective observational multicentre study	3981, of which 31 (0.78%) had a serious infection; of which 9/3981 had sepsis (0.22%)	Children presenting to General practitioner, pediatrician or the emergency department with acute illness	Serious bacterial infection: Pneumonia, meningitis, sepsis, pyelonephritis, osteomyelitis, bacterial gastroenteritis	Parental concern was described as “different illness” with the definition of a “statement by the parents that this illness was different from previous illnesses.” Presence of parental concern was associated with odds ratio for sepsis/meningitis of 70.5 (14.5–341.4); Sensitivity 77.8%, specificity 95.3%.

Four further studies reported original data, however none of these reported on diagnostic accuracy (Table 2). A secondary analysis of the 2007 Van den Bruel study investigated the role of clinician‘s gut feeling that something is wrong in the patients. Parental concern was identified as the strongest factor increasing the likelihood of clinician‘s gut feeling that something is wrong (univariate OR 26.93; 9.02 to 80.41, multivariate OR 36.26; 12.28 to 107.07) (38). However, the secondary analysis did not comment on the predictive accuracy of parental concern in relation to serious bacterial infection. Another study by Van den Bruel et al. (39) performed qualitative interviews with families and practitioners of 18 children hospitalized for severe bacterial infection with a mean age of 2.5 years (range 14 days to 11 years). Parents reported findings such as “*The moment he was sitting on my lap and suddenly collapsed, I was really frightened and came here immediately. At that moment I just knew it was more than just a cold*.” Van den Bruel et al. (39) referring to a 2-year-old child with sepsis. The authors concluded that parents have a high accuracy in describing the behavior of their children and to assess how the current behavior compares with normal behavior, and with behavior during previous illnesses. Another study performed qualitative interviews with General Practitioners to assess their attitudes in relation to recognizing children with meningitis and meningococcal septicemia (40). No patient data was assessed. Parental concern, and maternal “instinct” that the child was not right were mentioned as sometimes representing the only clues to a severe disease. Another study interviewed 95 parents from primary care inner city settings and performed focus groups to explore parental concerns about acute pediatric illness (41). Fever, cough and risk of meningitis emerged as key areas of concern. Sepsis was not specifically mentioned, however parents reported their fear that they may fail to recognize a life-threatening condition.

**Table 2:** Original studies reporting on parental concern as diagnostic measure of sepsis, not reporting on diagnostic accuracy of parental concern.

**References and country of enrolment**	**Study design**	***N* patients**	**Inclusion criteria**	**Outcomes in relation to parental concern**	**Comments**
Van den Bruel et al. (38), Belgium	Prospective observational multicentre study (121 physicians)	3980, of which 21 (0.53%) had a serious infection; of which 1/3980 had sepsis (0.02%)	Children presenting to General Practitioner, pediatrician or the emergency department with acute illness <5 day	Serious bacterial infection requiring hospital admission for >24 h: Pneumonia, meningitis, sepsis, pyelonephritis, osteomyelitis, bacterial gastroenteritis	Secondary analysis of the same dataset as published in Van den Bruel et al. (37) (Table 1). Parental concern was the strongest factor associated with a clinician's gut feeling that something was wrong OR 26.9, 9.0 to 80.4).
Van den Bruel et al. (39), Belgium	Single-site qualitative study. Interviews with parents and clinicians	No patients; Parents of 18 cases and 9 of the respective general practitioners interviewed	Families of children diagnosed with severe bacterial infection	N/A	Parental assessment that the disease is different from other diseases is highlighted.
Brennan et al. (40), United Kingdom	Qualitative prospective multi centre study. Semi-structured interviews.	26 General Practitioners	General practitioners of the area	N/A	General practitioners were interviewed to identify diagnostic approach in children with meningitis. Maternal intuition that “the child isn't quite right” and parental concern were stated as factors influencing medical decision making about the seriousness of infection.
Kai et al. (41), United Kingdom	Qualitative prospective study. Focus groups and interviews	95 parents	Parents registered at a General practitioner practice, at regional care facilities, and from parent groups	N/A	The concerns of parents related to lack of personal control and perceived threat by a serious illness (which may result in meningitis, disability, or death). Cough and fever were key concerns, and did not necessarily relate to severity. Parents were worried about failing to recognize a serious problem, and may struggle to define the severity of illness.

Six review articles were included which reported on parental concern in diagnosis of sepsis (Table 3). Two systematic reviews analyzed the findings from 36, and 35 articles, respectively, including 30 articles reporting on clinical features in relation to diagnosis of serious infection in children in developed countries (43, 47). The original study of Van den Bruel was the only study in both reviews which reported on parental concern to assist in the diagnosis of serious infection in children (37). While clinician‘s gut feeling that something was wrong performed better than parental concerns, the diagnostic accuracy of both parental concern and clinician‘s gut feeling outperformed most routinely used physiological or observational data in other studies. Four further non-systematic reviews and narratives were identified which listed parental concerns as a feature of children presenting with life-threatening infections or sepsis (42, 44–46). These articles did not use any original data to justify this but rather commented on the value of parental concern in the perspective by the authors.

**Table 3:** Reviews reporting on parental concern in relation to diagnosis of sepsis.

**References**	**Scope of review**	**Study design**	***N* studies**	**Inclusion criteria**	**Outcomes**	**Comments:**
Van den Bruel et al. (41)	Until June 2009	Meta analysis/review	30 studies included	Diagnostic accuracy studies on children to predict serious infection	Serious infection	1 study commenting on parental concern (37): High odds ratio for serious bacterial infection in presence of parental concern, and in presence of clinician instinct that something different
Niehues (42)	2009–29 Aug 2013	Narrative review	N/A	Pediatric fever management	N/A	Review on febrile infections in children. “Degree of parental concern” listed as a strong red flag
Thompson et al. (43)	Oct 2008. Update June 2009	Systematic review and validation of prediction rules	35 studies included in review	Prediction rules to identify children with serious infections in Emergency settings	Serious infection	1 study commenting on parental concern (37): Parental concern that the illness is different from previous illnesses (Likelihood ratio + 14) and the clinician's gut feeling that something is wrong (Likelihood ratio + 23)
Long (44)	N/A	Narrative review	N/A	N/A	N/A	Review on family stressors and perception during sepsis. Role of parents in recognizing altered behavior mentioned.
Printz (45)	N/A	Narrative review	N/A	N/A	N/A	Review on management of febrile illness. States that clinicians should listen to parental concerns as indicators of serious illness. Parents as experts of their child.
Yung (46)	N/A	Narrative review	N/A	N/A	N/A	Review on recognition of menincoccemia. Concerns of parents, relatives or friends are listed as clues for early recognition. Parents as best judges of the health of their children. Note of worry of relatives/friends which seems more extreme than presenting signs.

### Examples of Use of Parental Concern in Guidelines and Institutional Pathways

We assessed published and online available international pediatric sepsis guidelines pathways and guidelines in relation to the role of parental concern in the recognition of sepsis. In addition, we selected examples of published and online available institutional pathways designed for the recognition of sepsis in children (Table 4). The selection was limited to jurisdictions which has previously published on the state- or nation-wide implementation of sepsis bundles, specifically the United Kingdom, New York State in the United States, and New South Wales in Australia (14, 33–35).

**Table 4:** Selected examples of online available guidelines and pathways on pediatric sepsis recognition.

**Owner/institution/publication date**	**Website**	**Type**	**Target group**	**Location**	**Mention of parental concern**	**Role/utilization of parental concern**
American College of Critical Care Medicine, 2017	https://www.ncbi.nlm.nih.gov/pubmed/28509730	Guideline	Neonatal and pediatric	Endorsed by multiple national and international societies	Not mentioned	N/A
Surviving Sepsis Campaign, 2013	http://www.ncbi.nlm.nih.gov/pubmed/23361625	Guideline	Adult and pediatric	ED, PICU, and inpatient unit; Endorsed by multiple national and international societies	Not mentioned	N/A
National Institute for Health and Care Excellence (NICE), 2017	https://www.nice.org.uk/guidance/ng51/resources	Guideline and institutional pathway	Adult, pediatric, and neonate	ED, PICU, and inpatient unit; United Kingdom	Yes. –“ Pay particular attention to concerns expressed by the person and their family or carer” –“Parental or carer concern is important and should be acknowledged” –“Parent or carer concern that child is behaving differently from usual”	Used as screening “Could this be sepsis?” and as moderate-to-high risk criterium
United Kingdom Sepsis Trust, 2018	https://sepsistrust.org/professional-resources/clinical/	Pathway	Pediatric	ED, United Kingdom	Yes. - In <5 year and 5 to 12 year age groups: “Parents very worried”	Used as Amber Flag criterium
Department of Pediatrics, New York University, 2016	http://pediatrics.aappublications.org/content/pediatrics/137/3/e20144082.full.pdf	Pathway	Pediatric	Inpatient Unit, New York, United States	Not mentioned	N/A
Sepsis Kills, Clinical Excellence Commission, 2016	http://www.cec.health.nsw.gov.au/__data/assets/pdf_file/0008/343475/Pediatric-Sepsis-Pathway-Sept-2016-with-watermark.pdf	Pathway	Adult and Pediatric	ED, and inpatient unit, New South Wales, Australia	Yes -“High level parental concern “	Used as screening “Are you concerned your patient could have sepsis?”

*Current pediatric sepsis guidelines and selected examples of pathways from the United Kingdom, United States, and Australia are shown in reference to whether parental concern in sepsis recognition is mentioned*.

Two recent international guidelines pertinent to pediatric age groups, the American College of Critical Care Medicine Guidelines 2017 (48), and the 2013 Surviving Sepsis Campaign (49) guidelines, recommend that institutions implement sepsis screening tools. Parental concerns are not featured in these two guidelines. In contrast, the National Institute for Health and Care Excellence (NICE) (https://www.nice.org.uk/guidance/ng51/resources) and Sepsis Trust (Sepsis 6) (https://sepsistrust.org/professional-resources/clinical/) guidelines in the U.K. Both list parental concern as a feature of sepsis recognition. Two examples of institutional pathways from New York State, United States (http://pediatrics.aappublications.org/content/pediatrics/137/3/e20144082.full.pdf), and New South Wales, Australia (http://www.cec.health.nsw.gov.au/__data/assets/pdf_file/0008/343475/Pediatric-Sepsis-Pathway-Sept-2016-with-watermark.pdf), further illustrate that the utilization of parental concern varies in these pathways. Some list parental concern specifically as a feature that should support clinicians to think “Could this be sepsis,” others list it as one of several criteria prompting treatment, and some do not mention parental concern specifically.

## Discussion

In this literature review on parental concerns as a tool to assist in the recognition of sepsis, we identified a paucity of evidence to guide best practice. Only one study was found which assessed diagnostic accuracy of parental concerns in serious infections, suggesting superior performance of parental concern in comparison to routine physiological-based criteria. Several reviews highlighted the potential of parental concerns in recognizing children with life-threatening infections. Despite the fact that sepsis starts most commonly at home, the role of recognition of sepsis by parents to improve accuracy of early sepsis diagnosis represents a neglected field. Yet, several institutional and national sepsis quality improvement tools have embedded assessment for parental concerns as part of standardized sepsis screening. Our findings indicate an urgent need for well-designed diagnostic accuracy studies to define the value of assessing parental concerns in sepsis recognition in acute care settings. To the best of our knowledge this is the first review providing a comprehensive overview in the field.

The study by Van den Bruel prospectively assessed parental concerns, defined as a parental perception that the disease was different from previous illnesses (37). Despite the sample size of this well-designed study including 3982 visits to General Practitioners, Pediatricians, and Emergency Settings, the prevalence of serious infections was very low, and very few of the serious infections were reported as sepsis, hence resulting in low power for sepsis as an outcome. Despite these limitations, the performance of parental concern was clearly superior to routinely used physiological markers. The authors assessed as well the diagnostic value of clinician‘s gut feeling with serious infections and identified both parental and healthcare worker concerns as good predictors (38). Yet these findings may not necessarily reflect diagnostic performance in Emergency Department settings where patient acuity is higher. In addition, in larger Emergency Departments, a majority of parents already has gone through a selection process by community physicians and parental concern potentially may be less discriminative, restricting the generalizability of the findings by Van den Bruel.

Qualitative studies have shown that parents of children presenting with severe infections report on changes in their child‘s observed behavior ranging from altered crying or mentation, to moaning or inconsolability (43, 47), highlighting the role of parents as experts of their child‘s behavior. The value of parental involvement in healthcare decision-making and provision has been increasingly recognized in areas other than sepsis, building up on the unique position of parents being experts of their child. Structured parental education on early recognition of severe infections has become standard in the management of oncologic children and children discharged with indwelling medical devices (50, 51). While the setting fever and neutropenia may allow easier operationalization than the more vague concept of sepsis, parents of immunosuppressed children are empowered to raise concerns and are often considered part of the experts in making informed decisions about best care to their children. Importantly, in resource poor settings, maternal education has been demonstrated to lead to reduced infection-related mortality during childhood (28), likely through improved prevention and faster recognition of disease leading to earlier treatment.

At present, several campaigns incorporate parental education and empowerment on sepsis, for example the Sepsis Assessment and Management (SAM) tool in the United Kingdom (http://www.southdevonandtorbayccg.nhs.uk/your-health/Documents/sam-sepsis-leaflet.pdf). The Public Health England and the UK Sepsis Trust jointly lead a campaign to improve parental awareness and knowledge of sepsis (http://www.independent.co.uk/life-style/health-and-families/health-news/sepsis-campaign-nhs-jeremy-hunt-children-condition-what-are-symptoms-signs-child-health-a7476426.html. In New York State, education of children on sepsis has become mandatory as part of the Rory Staunton regulations (http://www.nysed.gov/curriculum-instruction/sepsis). Kerkhof et al analyzed data from over 6,000 children and assessed the predictive performance of NICE criteria and suggested future iterations should consider parental concern (52). The more recent NICE guidelines on the recognition of sepsis in children include parental concerns.

Several challenges may arise when including parental concerns as a tool to recognize sepsis: First, the prevalence of sepsis is very low across most pediatric Emergency Departments where hundreds of children present daily with non-septic febrile infections. At the same time, most parents of children with acute infections attending Emergency Departments (rather than General Practitioners) may have substantial concerns. Second, the parental understanding on the possible life-threatening nature of a disease is likely influenced by common belief (“cough” or “fever” is dangerous) rather than specific features of disease. Yet, the paternalistic approach assuming that medical practitioners and Early Warning Tools (19, 53, 54) perform superior to parents may falls short of daily challenges in the provision of medical care: the level of experience of many doctors involved in initial patient assessment may be low, and during busy periods medical staff may not always have sufficient time to assess all patients thoroughly.

Third, the need for, and the benefit of antimicrobial stewardship may not be directly evident to parents who present with a child with a mild disease yet are concerned this could be sepsis—some parents may request antibiotic therapy to prevent or treat potential progression to a severe disease (25). Indeed, a cluster randomized controlled factorial trial evaluated a brief intervention to elicit parental concern combined with safety net advice and found increased antibiotic prescribing in children allocated to this arm (55). The study findings imply that over-prescription of antibiotics needs to be considered as a balancing measure when designing interventions focusing on parental concern. Forth, a study in east Africa (56) investigated the educational background of parents in relation to presentations for pediatric illness and demonstrated that the majority of the parents had very limited knowledge of their children's health problems as assessed in the study. This illustrates the considerable cultural and educational challenges in applying parental concern-based approaches in low income settings. Finally, individual parents may have different perceptions and approaches to risks related to infectious diseases and treatment (57).

Future studies should prospectively assess the diagnostic accuracy of parental assessment of infectious disease severity across low, middle, and high income settings with particular focus on sepsis and septic shock. Further information is needed to specifically analyze the benefit of providing targeted sepsis education to parents. Given the unique role of parents as experts of their child, such interventions have in principle a substantial potential to enhance the diagnostic performance of screening for sepsis as part of a rule-in approach. Yet, such approaches needs to be balanced against the risk that creating community awareness of sepsis may lead to excessive consumption of healthcare resources for children with mild infections, and lead to unnecessary treatment. Hence, while it may be beneficial for clinicians to include questions pertinent to the level of parental concern in their assessment of the acutely ill child evaluated for infection, clinicians should be empowered to consider diagnoses other than sepsis and make an informed decision to rule-out sepsis if such is considered unlikely (25). Parents should be considered key partners in safety netting of such children where sepsis was ruled out—ongoing observation at home with the availability for prompt representation to reassess may potentially reduce adverse outcomes from late sepsis presentations.

### Limitations

Several limitations of this review need to be considered. First, only one original study was identified reporting on the diagnostic accuracy of parental concerns in the recognition of sepsis, with a very small number of children meeting the outcome, and a meta-analysis could not be performed. Second, the design and quality of included articles was variable, ranging from original quantitative studies, original qualitative studies, and high quality systematic reviews to non-systematic narratives. In view of the lack of data, the review was extended to include original studies not reporting on diagnostic accuracy and reviews referring to parental concern in children with sepsis. Third, only abstracts leading to full text publications in English were considered, and this may have further reduced the yield of the search. Finally, the overview of institutional sepsis pathways represents a selection pertinent to recently published international sepsis guidelines, and jurisdictions who have reported on sepsis pathway implementation.

## Conclusion

In conclusion, this review identified a paucity of data analyzing the role of parental concerns in recognizing sepsis. Several guidelines and institutional protocols emphasize the importance of listening to parents and utilize parental concerns as one of the trigger criteria for sepsis recognition. Parental concern needs to be considered to improve accuracy in recognizing life-threatening infections in children. Education on utilizing parental concerns to recognize sepsis has the potential to lead to better outcomes in paediatric sepsis. However, understanding parental perceptions of sepsis, and parental decision-making in seeking advice is urgently needed to inform on optimal design of education campaigns, and to assist in the design of sepsis recognition bundles. Prospective studies are needed testing the sensitivity, specificity, and negative and positive predictive value of parental concerns in sepsis in various settings, while assessing the potential impact on antibiotic and health care resource use.

## Author Contributions

AH and LS designed the study. All abstracts were checked by AH and LS. AH and LS went through each of the full texts. JL assisted in study design, review of results, and manuscript writing. LS and AH wrote the first draft of the manuscript. All authors contributed to manuscript revision and approved the final version.

### Conflict of Interest Statement

The authors declare that the research was conducted in the absence of any commercial or financial relationships that could be construed as a potential conflict of interest.
