# Supplementary material for: The Role of Parental Concerns in the Recognition of Sepsis in Children: A Literature Review
Source: Front Pediatr. 2019 May 3;7:161. doi: 10.3389/fped.2019.00161 (PMC6509218; doi:10.3389/fped.2019.00161)
Supplement: Supplementary file 1 [file Table_1.docx]

**Supplementary Material**

**The role of parental concerns in the recognition of sepsis in children: A literature review**

Amanda Harley^1,2,3^, Jos M Latour, PhD^4^, Luregn J Schlapbach, MD^1,2,5*^

**Supplementary Table 1**

| **Database** | **Date searched** | **Search terms** | ***n*** | **Limits applied** | ***n*** | **Title screened**  ***n* remaining** |
| --- | --- | --- | --- | --- | --- | --- |
| CINAHL  (EBSCO HOST) | 1/9/19 | (Concern OR worry OR fear) AND (infant OR pediatric OR paediatric OR child OR neonate OR childhood AND sepsis or septic or severe sepsis or septic shock OR bacteremia OR severe infection OR SIRS AND parent OR family OR caregiver OR mother OR Father) All single searches according to category then combined= (Concern OR worry OR fear) AND (S5 AND S6 AND S7 AND S8) | 58 | Boolean included | 58 | 27 |
| PubMed | 1/9/19 | ("parent*"[MeSH Terms] OR "parents"[All Fields] OR "parent"[All Fields]))) AND (("Concern"[Journal] OR "Concern (Anaheim)"[Journal] OR "concern" OR "fear"[All Fields] OR fear))) AND (("pediatrics"[MeSH Terms] OR "pediatrics" OR "neonatal"[All Fields] OR "pediatric"[All Fields] OR child*) AND ("sepsis"[MeSH Terms] OR "sepsis" OR "bacteremia" OR "septic shock" [All Fields]) | 31 | 1990-2018 | 23 | 4 |
| Medline (OVID) | 1/9/19 | 1     Sepsis/ (52849)  2     [sepsis.tw](http://sepsis.tw). (82629)  3     1 or 2 (108560)  4     exp Infant/ (1058439)  5     (infant* or infancy or newborn* or baby* or babies).tw. (526412)  6     exp Child/ (1757559)  7     (child* or schoolchild* or school age* or preschool* or kid or kids or toddler*).tw. (1237647)  8     Adolescent/ (1848477)  9     (adoles* or teen* or boy* or girl*).tw. (428557)  10     Minors/ (2437)  11     Puberty/ (12703)  12     (minor or minors or pubert* or pubescen* or prepubescen*).tw. (239943)  13     exp Pediatrics/ (52847)  14     (pediatric* or paediatric*).tw. (293599)  15     or/4-14 (3968065)  16     3 and 15 (31381)  17     parents/ or fathers/ or mothers/ or single parent/ (94363)  18     (parent* or mother* or father*).tw. (536631)  19     or/17-18 (558095)  20     attitude/ or attitude to health/ or health knowledge, attitudes, practice/ or "treatment adherence and compliance"/ or optimism/ or pessimism/ (210276)  21     (concern* or attitud*).tw. (629240)  22     or/20-21 (775312)  23     19 and 22 (55451)  24     ((parent* or mother* or father* or carer*) adj3 (concern* or attitud* or perspective*)).tw. (10451)  25     Parents/px [Psychology] (23240)  26     or/23-25 (72813)  27     16 and 26 (160) | 130 | English language. | 130 | 16 |
